# Risk of Switch to Mania/Hypomania in Bipolar Depressive Patients Treated with Antidepressants: A Real-World Study

**DOI:** 10.34133/hds.0209

**Published:** 2025-06-03

**Authors:** Lei Feng, Weiwei Wang, Can Yin, Jing Li, Xinwei Zhang, Xiaotian Chang, Zizhao Feng, Mui Van Zandt, Seng Chan You, Sarah Seager, Christian Reich, Siyan Zhan, Feng Sun, Gang Wang

**Affiliations:** ^1^ Beijing Key Laboratory of Mental Disorders, National Clinical Research Center for Mental Disorders and National Center for Mental Disorders, Beijing Anding Hospital, Capital Medical University, Beijing 100088, China.; ^2^ Department of Epidemiology and Biostatistics, School of Public Health, Peking University, Beijing 100191, China.; ^3^ Key Laboratory of Epidemiology of Major Diseases (Peking University), Ministry of Education, Beijing 100191, China.; ^4^ Real World Solutions, IQVIA, Durham, NC 27703, USA.; ^5^ Observational Health Data Sciences and Informatics, New York, NY 10032, USA.; ^6^ SmindU Medical Science & Technology Co., Ltd., Beijing 1000125, China.; ^7^ Department of Counseling and Clinical Psychology, Teachers College, Columbia University, New York, NY 10027, USA.; ^8^ Department of Biomedical Systems Informatics, Yonsei University College of Medicine, Seoul 03722, Republic of Korea.

## Abstract

**Background:** The use of antidepressants in the treatment of bipolar depression remains controversial due to concerns about their potential to induce mood polarity switches. This multinational observational study aims to examine the association between the use of antidepressants and the risk of hypomanic/manic switch among bipolar depressive patients. **Methods:** Four electronic health record databases (IQVIA Disease Analyzer Germany, IQVIA Disease Analyzer France, IQVIA US Hospital Charge Data Master, and Beijing Anding Hospital) and one administrative claims database (IQVIA US Open Claims) were analyzed, and the study period covered from January 2013 until December 2017. Treatment patterns of patients with bipolar depression were collected. The hazard ratio (HR) was calculated by comparing the incidence of hypomanic/manic switch in patients who received antidepressants (AD group) with that in those who did not receive any antidepressant (non-AD group) in 730 days after the date of the first diagnosis of bipolar depression. **Results:** The analysis included a total of 122,843 patients from the 5 databases; 60.6% of them received antidepressants for bipolar depression. Across the 5 data sources, the mean age at index date ranged from 37.50 (15.72) to 52.10 (16.22) years. After controlling potential confounders by propensity score matching, the AD group’s manic switch risk was not significantly higher than the non-AD group’s (HR 1.04 [95% CI, 0.96 to 1.13]; *P* = 0.989). Additionally, no statistically significant difference was observed between patients prescribed antimanic drugs and those who were not (HR 0.69 [95% CI, 0.38 to 1.25]; *P* = 0.535). **Conclusions:** This study indicated that antidepressants were widely used in clinical settings for managing bipolar depression. The use of antidepressants was not associated with the risk of mania/hypomania switch when compared to non-antidepressants treatment. Therefore, antidepressants could be considered a treatment option for bipolar depression.

## Introduction

Bipolar disorder is a chronic and recurrent affective disorder distinguished by alternating episodes of elevated mood and depression, which often result in functional impairments and rank among the leading causes of disability in working-age adults [1]. A predominance of depression characterizes the long-term course of bipolar disorder, while at least one previous manic, hypomanic, or mixed episode is required to define the diagnosis of bipolar disorder [2–4]. Throughout the course of the illness, depressive episodes account for 72% of the total duration, and patients often require an extended period to achieve remission from these episodes [5,6]. Even with comprehensive treatment, patients with bipolar disorder are in a state of depression for 3 times more than they are in mania [7]. Therefore, treatment of depression remains a major challenge for the clinical management of bipolar disorder.

Compared with the manic episode, there are fewer effective treatments available for the depressive episode. The neurobiological mechanism underlying bipolar disorder remains insufficiently understood, hindering the development of targeted and precise therapies. So far, while 5 atypical antipsychotics (quetiapine, lurasidone, cariprazine, lumateperone, and olanzapine) have been approved for bipolar depression, their side effect profiles (e.g., somnolence, sedation, weight gain, and metabolic risks) often limit their use in clinical settings compared to antidepressants [8]. Additionally, the efficacy of most mood stabilizers and atypical antipsychotics in managing depressive episodes has not been well established. As a result, antidepressants are often prescribed concurrently with mood stabilizers or atypical antipsychotics for patients with bipolar depression [9–11]. Antidepressants are considered adjunctive therapeutic options in bipolar depression patients who are resistant to mood stabilizers and antipsychotics [8,12,13]. However, the administration of antidepressants during bipolar depressive episodes is debated due to the potential risk of causing a switch to hypomania/mania [13–15]. A primary concern about a switch to mania is that manic episodes coupled with impaired judgment often result in disruptive or aggressive behaviors adversely affecting important areas of functioning, such as relationships and employment, and may require hospitalization [16]. Furthermore, mixed episodes, characterized by simultaneous symptoms of mania and depression, are associated with heightened emotional dysregulation, increased impulsivity, and a higher risk of adverse outcomes, including self-harm and hospitalization [17]. Moreover, antidepressants may alter or exacerbate the clinical course of the illness for months or years [18]. While the latest network meta-analysis suggested no significant difference in switching to mania between antidepressants and placebo, other works showed mixed results [12,13,19–25]. Such discrepancy could potentially be the result of the heterogeneity among participants and treatments in individual clinical trials. For example, variations in treatment protocols, such as differences in treatment duration or the use of adjunctive therapies, may contribute to inconsistencies. Specifically, the randomized clinical trial (RCT) durations ranged from 2 to 16 weeks in the network meta-analysis by Yildiz et al. [12], whereas other systematic reviews included studies with no duration restrictions [13], a minimum of 4 months [22], or continuation for at least 6 months [24]. Understanding the potential risks of antidepressants in bipolar depressed patients is crucial, particularly when considering severe outcomes such as sudden mania. However, studying these events in RCTs is challenging due to the inherent limitations of RCTs, including small sample sizes and strict inclusion criteria that often exclude patients who represent the broader, more diverse population in clinical practice [26]. For example, many antidepressant trials exclude patients at higher risk for mania, such as those with a coexisting substance abuse or those who have been hospitalized for manic episodes, which restricts the generalizability of the results [27,28]. In contrast, electronic health record data can circumvent these limitations and provide real-world evidence from a general patient population, helping to fill the gap left by these trials. In this retrospective cohort study, we aimed to evaluate the role of antidepressants in the risk of switching to hypomania/mania among patients with bipolar depression.

## Methods

### Study design and data sources

This retrospective cohort study used 4 electronic health record (EHR) databases and one administrative claims database from the United States, Germany, France, and China. EHR databases originate from clinical practice and provide detailed documentation of patient encounters, including diagnoses, prescribed medications, laboratory results, and clinical notes recorded during visits. In contrast, administrative claims databases are primarily generated for billing and reimbursement purposes, capturing information on healthcare services provided and associated costs. Claims data typically include diagnostic and procedure codes, prescription records, and demographic details but lack the granular clinical observations and laboratory results present in EHR databases. We conducted propensity score matching within each database to control potential confounding. All the databases were standardized to Observational Medical Outcomes Partnership Common Data Model (OMOP CDM version 5.3), which maps international coding systems into standard vocabulary concepts [29]. Data nodes keep protected health information within their firewalls to maintain privacy. Queries are distributed and executed locally, with only aggregated results returned centrally [30]. A protocol for this study was approved by all data owners. Thus, the study is exempted from the requirement for ethics approval. Data from 2013 January 1 through 2019 December 31 were included in the current study, so the analysis by large covered modern antidepressants rather than older generations (e.g., tricyclics or monoamine oxidase inhibitors). The introduction of the databases and their characteristics are shown in Table S1.

### Study population

The study population included patients with outpatient visits who were first diagnosed with bipolar depression (bipolar disorder, current episode depressed) between 2013 January 1 and 2017 December 31. The time-at-risk window for mania was defined as from the index date (i.e., the date of outpatient visit with the first bipolar disorder diagnosis) to the index date + 730 days to ensure a sufficient length of observation. Excluded patients had any diagnoses of schizophrenia spectrum disorder, nonschizophrenia psychotic disorders, dementia, neurodegenerative disease, or other specified mental and behavioral disorders at any time prior to the index date.

Bipolar depression diagnoses were identified by ICD-10 codes (e.g., F31.3, F31.4, and F31.5, Table S2), and the antidepressant prescription was identified by RxNorm codes (Table S3). For the contributing data assets, all the individuals satisfying the eligibility criteria for the study were included.

### Exposure

Patients must be diagnosed with bipolar depression and prescribed antidepressants during the same visit. The following 2 study cohorts were defined: (a) those who received antidepressants (AD group) and (b) those we did not receive any antidepressants (non-AD group).

### Outcomes

The outcome of interest is the first occurrence of the switch to mania or hypomania, which was defined as the occurrence of the diagnosis of hypomania and mania (ICD-10 codes: F30.0-F30.2, F30.8-F30.9, and F31.0-F31.2, Table S2) during a defined time-at-risk window of 730 days. The decision to use a 730-day (2-year) follow-up period was based on the clinical understanding that bipolar spectrum disorders, including Bipolar II disorder, are characterized by chronic, fluctuating periods of hypomania and depression that typically persist for at least 2 years [31].

### Statistical analysis

Regularized logistic regression [32,33] was used to estimate the propensity scores, which used all the available baseline patient characteristics including demographic characteristics (e.g., age in 5-year bands, sex), medical history (e.g., chronic liver disease, renal impairment, diabetes mellitus, hyperlipidemia, hypertensive disorder, obesity, and gastrointestinal hemorrhage), and medication (e.g., psychostimulants, opioids, antiepileptics, antipsychotics, agents acting on the renin–angiotensin system, antithrombotic agents, beta blockers, calcium channel blockers, diuretics, acid-suppressive agents, antidiabetic medications, and lipid-lowering agents) in each database. The rationale behind the potential confounding was the presence of medical comorbidities in patients with bipolar disorder, such as metabolic syndrome (37%), obesity (21%), and type 2 diabetes (14%), which were associated with both treatment and outcome [31]. Stimulants were considered as risk factors for affective switch [18]. Additionally, adverse events (e.g., weight gain, chronic kidney disease, and elevation in liver function test) associated with pharmacological treatments could be related to the treatment options and impact the outcome [31]. All medications should be prescribed within 7 days after the index date to be considered as initial treatment pattern. The study populations were matched using a one-to-one greedy caliper propensity score matching technique with a caliper width of 0.02 [34]. Greedy caliper matching, a popular propensity score matching technique, orders the treated subjects and sequentially matches each treated subject to an untreated (control) subject whose propensity score falls within a predefined caliper width [35]. Propensity score matching has been demonstrated to be a reliable method for providing excellent covariate balance in most circumstances [36]. Cox proportional hazard regression models were used to estimate the association between exposures and outcomes. Patients were censored when they were no longer observed in the database. Those who discontinued or switched their allocated medications within the first year were included in the primary analysis. With regard to the AD group, a subgroup analysis was conducted to compare the risk of hypomanic/manic switch between the AD monotherapy group and the AD concomitant with stabilizers/antipsychotics group. Considering the challenges associated with accurately capturing concomitant medication data, especially considering the potential underreporting in administrative claims databases, we performed subgroup analyses using 4 EHR databases to ensure the robustness of our findings. We conducted negative control outcome experiments to evaluate potential residual confounding in the analyses, under the assumption that the null hypothesis of no effect would hold true for these controls. A total of 33 negative control outcomes were identified based on their lack of biological plausibility to be influenced by the exposure of interest (Table S4). The results of these experiments showed no significant associations, supporting the robustness of our primary analysis. A random-effects meta-analysis was performed to calculate the summary hazard ratio (HR), synthesizing effect estimates across the databases [37].

## Results

### Patient characteristics

**Fig. 1.**
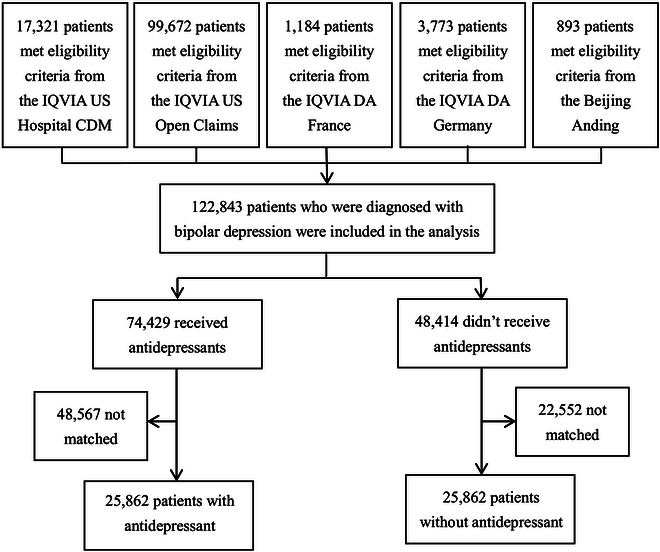
Study flowchart of patients with bipolar depression whether given antidepressants or not.

A total of 122,843 patients were included in the analysis, with 99,672 from Open Claims, 17,321 from Hospital CDM, 3,773 from DA Germany, 1,184 from DA France, and 893 from Beijing Anding Hospital database (Fig. 1). Table 1 shows the demographic characteristics of eligible patients across the 5 data sources. Male patients accounted for about one-third of the total number of bipolar depression patients. Across the 5 data sources, the mean age at index date ranged from 35.56 (±14.86) to 53.11 (±15.27) years.

**Table 1.** Baseline characteristics of patients with bipolar depression whether given antidepressants or not across the 5 data sources

	IQVIA DA France		IQVIA DA Germany		IQVIA US Hospital CDM		IQVIA US Open Claims		Beijing Anding		Pooled from 5 databases	
	AD	Non-AD	AD	Non-AD	AD	Non-AD	AD	Non-AD	AD	Non-AD	AD	Non-AD
Sample size	502	682	1,077	2,696	3,072	14,249	69,274	30,398	504	389	74,429	48,414
Age, mean (SD)	52.67 ± 14.37	50.55 ± 15.26	53.11 ± 15.27	51.70 ± 16.57	49.2 ± 14.46	45.74 ± 15.14	44.9 ± 16.22	46.90 ± 18.71	39.0 ± 16.21	35.56 ± 14.86	47.80 ± 15.31	46.21 ± 16.14
Male, *n* (%)	188 (37.5)	201 (29.5)	412 (38.3)	1,036 (38.4)	819 (26.7)	4,161 (29.2)	19,840 (28.6)	12,016 (39.5)	208 (41.3)	166 (42.7)	21,467 (28.8)	17,580 (36.3)
Previous manic episodes, *n* (%)	7 (1.4)	22 (3.2)	122 (11.3)	296 (11)	195 (6.4)	928 (6.5)	6,740 (9.7)	2,982 (9.8)	135 (26.8)	132 (33.9)	7,199 (9.6)	4,360 (9)
Mood stabilizers, *n* (%)	133 (26.5)	196 (28.7)	370 (34.4)	700 (26)	1,454 (47.3)	653 (4.6)	35,676 (51.5)	5,393 (17.7)	307 (60.9)	233 (59.9)	37,940 (51.0)	7,175 (14.8)
Lithium, *n* (%)	NA	NA	170 (15.8)	357 (13.2)	368 (12)	215 (1.5)	NA	NA	172 (34.1)	160 (41.1)	710 (15.3)	732 (4.2)
Divalproex, *n* (%)	83 (16.5)	118 (17.3)	102 (9.5)	175 (6.5)	544 (17.7)	254 (1.8)	12,130 (17.5)	1,869 (6.2)	242 (48)	169 (43.4)	13,101 (17.6)	2,585 (5.3)
Lamotrigine, *n* (%)	40 (8)	63 (9.2)	130 (12.1)	225 (8.4)	685 (22.3)	218 (1.5)	26,283 (37.9)	3,633 (12)	24 (4.8)	29 (7.5)	27,162 (36.5)	4,168 (8.6)
Carbamazepine, *n* (%)	21 (4.2)	25 (3.7)	20 (1.9)	41 (1.5)	100 (3.3)	48 (0.3)	2,979 (4.3)	331 (1.1)	NA	NA	3,120 (4.2)	445 (0.9)
Olanzapine/fluoxetine (Symbyax) or lurasidone or quetiapine or cariprazine, *n* (%)	51 (10.2)	80 (11.7)	279 (25.9)	557 (20.7)	964 (31.4)	343 (2.4)	31,305 (45.2)	4,143 (13.6)	191 (37.9)	145 (37.3)	32,790 (44.1)	5,268 (10.9)
Antipsychotics, *n* (%)	168 (33.5)	244 (35.8)	409 (38)	807 (29.9)	2,026 (66)	1,074 (7.5)	49,524 (71.5)	7,618 (25.1)	317 (62.9)	257 (66.1)	52,444 (70.5)	10,000 (20.7)
First-generation antipsychotics, *n* (%)	28 (5.6)	46 (6.7)	41 (3.8)	62 (2.3)	693 (22.6)	499 (3.5)	7,689 (11.1)	1,106 (3.6)	52 (10.3)	41 (10.5)	8,503 (11.4)	1,754 (3.6)
Second-generation antipsychotics, *n* (%)	156 (31.1)	223 (32.7)	392 (36.4)	785 (29.1)	1,823 (59.3)	705 (5)	48,145 (69.5)	7,058 (23.2)	307 (60.9)	252 (64.8)	50,823 (68.3)	9,023 (18.6)

AD, antidepressants; NA, the proportion is less than 0.1%.

### Treatment patterns

For the initial treatment pattern, 60.6% of patients in the aggregated 5 databases were treated with antidepressants. The proportion varied significantly by database, ranging from 17.7% in Hospital CDM to 69.5% in Open Claims. Mood stabilizers were prescribed to 60.5% of the patients in BJ Anding but only 12.2% in Hospital CDM. Antipsychotics were used in 64.3% of patients in BJ Anding, with second-generation antipsychotics accounting for the majority (62.6%). In addition, the corresponding percentages for each database, including the use of Food and Drug Administration (FDA)-approved drugs (i.e., olanzapine/fluoxetine, lurasidone, quetiapine, and cariprazine) for bipolar depression, are presented in Table 2.

**Table 2.** Treatment patterns of patients with bipolar depression across the 5 data sources

	IQVIA DA France	IQVIA DA Germany	IQVIA US Hospital CDM	IQVIA US Open Claims	Beijing Anding	Pooled from 5 databases
Sample size	1,184	3,773	17,321	99,672	893	122,843
Antidepressants, *n* (%)	502 (42.4)	1,077 (28.5)	3,072 (17.7)	69,274 (69.5)	504 (56.4)	74,429 (60.6)
Mood stabilizers, *n* (%)	329 (27.8)	1,070 (28.4)	2,107 (12.2)	41,069 (41.2)	540 (60.5)	45,115 (36.7)
Prescription of drug approved by FDA for bipolar depression, *n* (%)	131 (11.1)	836 (22.2)	1,307 (7.5)	35,448 (35.6)	336 (37.6)	38,058 (31)
Antipsychotics, *n* (%)	412 (34.8)	1,216 (32.2)	3,100 (17.9)	57,142 (57.3)	574 (64.3)	62,444 (50.8)
Second-generation antipsychotics, *n* (%)	379 (32)	1,177 (31.2)	2,528 (14.6)	55,203 (55.4)	559 (62.6)	59,846 (48.7)

Baseline characteristics of the study population, both before and after propensity score matching, were presented for each data source in Tables S5 to S9. After propensity score matching, 25,862 propensity-matched pairs exhibited balanced baseline characteristics between the antidepressant group and the non-antidepressant group within each database.

### Overall effects of antidepressants on risk of mania

**Fig. 2.**
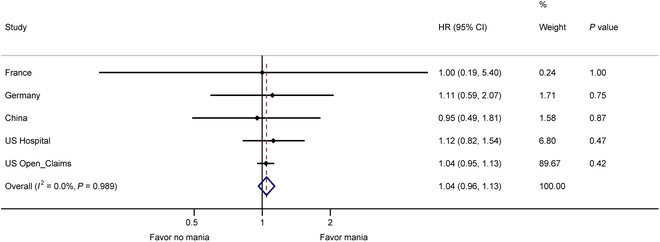
Risk of mania/hypomania switch at 730 days.

The prevalence of mania switch was 5.2% overall, and 5.6% and 4.9% within the AD and non-AD groups, respectively. During the observation period, the risk of switch to mania was not significantly different between the AD group and the non-AD group in the US Open Claims (HR, 1.04 [95% CI, 0.95 to 1.13]; *P* = 0·42), US Hospital CDM (HR, 1.12 [95% CI, 0.82 to 1.54]; *P* = 0.47), Germany (HR, 1.11 [95% CI, 0.59 to 2.07]; *P* = 0.75), France (HR, 1.00 [95% CI, 0.19 to 5.40]; *P* = 1.00), and China (HR, 0.95 [95% CI, 0.49 to 1.81]; *P* = 0.87). Overall, the 2-year incidence of mania switch was 27.24 per 1,000 person-years in the AD group and 25.37 per 1,000 person-years in the non-AD group. The meta-analysis showed no significant difference in overall mania switch between the AD group and the non-AD group (pooled HR, 1.04 [95% CI, 0.96 to 1.13]; *P* = 0.989; Fig. 2). Kaplan-Meier curves were presented in the Supplementary Materials (Figs. S1 to S5).

**Fig. 3.**
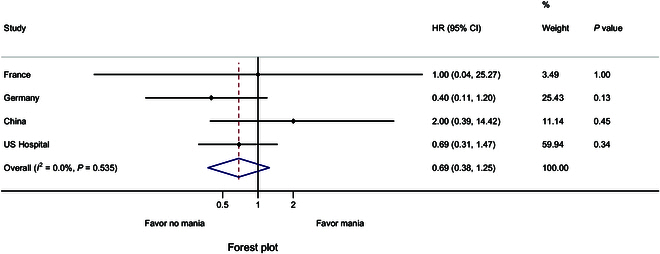
Forest plot of the hypomanic/manic risk between the antidepressants administered concomitantly with and without stabilizers/antipsychotics in the subgroup analysis.

When comparing antidepressant monotherapy to antidepressants combined with antimanic drugs (mood stabilizers or antipsychotics), the pooled HR of hypomanic/manic risk was 0.69 (95% CI, 0.38 to 1.25; *P* = 0.535; Fig. 3).

## Discussion

In this large-scale observational study that included over a hundred thousand patients with bipolar depression, nearly half of them were prescribed antidepressants. The results showed a neutral risk of inducing the switch to hypomanic/mania between the AD group and the non-AD group among patients with bipolar depression. As noted in current reviews, both the safety and efficacy of antidepressant on bipolar depression continue to provide mixed results [18,38,39].

In line with previous studies [38,40], the proportion of bipolar depressive patients who received antidepressants as initial treatment was relatively high (60.59%) despite the fact that antidepressants were not recommended as the first-line treatment for those conditions in clinical guideline and recommendations [8,41]. Similarly, a recent study using nationally representative data from the National Ambulatory Medical Care Survey conducted in the United States reported that prescription of antidepressants occurred in 57.5% of visits for bipolar disorder in the 2013 to 2016 period [9]. A retrospective cohort study using data from nationwide Finnish registers also found that the prevalence of antidepressant use was 40.8% at the 3-month time point after diagnoses of bipolar disorder [42]. Several possible explanations could be proposed. First, the use of lithium decreased substantially because it is commonly regarded as a difficult medication to manage, as the dose needs to be titrated [43]. Additionally, it requires regular monitoring of serum lithium levels attributable to the potential negative health consequences [44,45]. Second, most patients with bipolar disorder spent the majority of their course in depressive episodes, and depressive symptoms had more significant negative impacts on psychosocial functioning and suicide risk than hypomanic/manic symptoms [7,46–48]. These clinical features might prompt practitioners to prescribe antidepressants. Besides, the adjunctive use of antidepressants with a mood stabilizer or an atypical antipsychotic was demonstrated to be effective [13].

In this study, the AD group showed a higher risk of switching to mania compared to the non-AD group, although the difference was not statistically significant. A meta-analysis of 15 trials conducted by Sidor and Macqueen [21] that included patients with acute bipolar depression (typically observed over 6 to 10 weeks) also showed no significant association between AD and a higher risk of manic switch. The results were in accordance with the results of 3 previous systematic reviews [22,27,49] that reported similar rates of treatment-emergent switch to mania/hypomania in the antidepressant group and the placebo group with or without an adjunctive mood stabilizer. In a meta-analysis by Zhang et al. [19], short-term (4 to 12 weeks) and long-term (26 to 50 weeks) use of antidepressants, primarily with concomitant medication (except in one RCT), for the treatment of bipolar disorder were not associated with a significantly greater risk of switching to mania in RCTs. Similarly, a recent systematic review [50] that included 13 RCTs (interquartile range, 6 to 8 weeks) investigating the safety of antidepressants (i.e., imipramine, escitalopram, fluoxetine, olanzapine/fluoxetine, venlafaxine, bupropion, fluvoxamine, clomipramine, and tranylcypromine) also found that there was no difference in the rate of treatment-emergent affective switch between the treatment group and the placebo group.

It is true that there are a few studies presenting different results. For instance, a previous systematic review assessing the efficacy and safety of adjunctive therapies for bipolar depression, which involved the combination use of a second-generation antidepressant and mood stabilizer or atypical antipsychotic, reported a higher risk of treatment-induced mania or hypomania in a 52-week extension period (1.774 [1.018 to 3.091], *P* = 0.043), though the association was not significant in acute treatment (0.926 [0.576 to 1.491], *P* = 0.753) [13]. Additionally, 3 observational studies with a relatively limited sample size of each antidepressive agent suggested that antidepressants increased the risk of switch to mania [15,51,52].

However, several potential mechanisms may account for the inconsistency. First, participants in this study were all outpatients with less severe symptoms and previous studies included inpatients with more severe symptoms who might respond differently to antidepressants. Second, the null association might be attributable to the lack of information on bipolar disorder subtypes, as the risk of inducing mania/hypomania was higher in bipolar I disorder than in bipolar II disorder [53,54]. In addition, the observed marginally higher rate of switch to mania in the AD group could be due to the unpredictably fluctuant nature of mood state in bipolar disorder, rather than being directly attributed to drug therapy [55–57].

Our result indicated that the use of antidepressants did not significantly elevate the risk of hypomanic/manic switch in real-world clinical practices. Also, given the many side effects of mood stabilizers and second-generation antipsychotics that jeopardize the patients’ compliance and expected response, we propose that after adequate assessment of the patients’ condition, antidepressants may be a beneficial treatment for bipolar depression.

Our study highlights the potential of real-world EHR data in investigating the risks associated with antidepressant use in bipolar depression, a topic that has traditionally been challenging to explore in RCTs due to limitations such as difficulty in capturing outcome events of interest. In contrast to previous studies, our research specifically focuses on outpatients, who represent a significantly larger population compared to hospitalized patients. While our findings are of immense clinical importance, they also underscore the need for further research. Future studies would be valuable to explore how factors such as bipolar disorder subtypes, depression severity and duration, antidepressant type, treatment duration, dosage, and temporal variables may influence the outcomes.

### Strengths

This study has several strengths. First, unlike previous research that used highly selective samples in RCTs that excluded patients with severe symptoms, the sample was large, population-based, and generally representative. Second, this study included 5 administrative claim data and EMR data from 4 different countries that had been converted to OMOP CDM version 5.3. The OMOP CDM unifies data from heterogeneous data sources with respect to terminologies and overall structure, enabling integrated analysis across global healthcare systems. Besides, this is the first time that psychiatric data in China have been converted to OMOP and used in an international collaboration.

### Limitations

Several limitations of this study also need to be considered. First, a major limitation of the current study is the potential bias of confounding by indication although propensity score matching has been conducted to control confounders. Patients with a mania-predominant polarity, history of treatment-emergent mania or hypomania, current or predominant mixed features, or recent rapid cycling are less likely to be prescribed antidepressants [55,58]. Second, the treatment outcome of antidepressants may be underestimated because practitioners in real-world settings usually receive limited research training to assess manic or hypomanic episodes. Third, no instrument was used to spot treatment-emergent hypomanic/manic symptoms closely and regularly [59], thus underestimating the onset of the switch to hypomanic/mania. However, there may be an overestimation of mania switch because of the difficulty differentiating between hypomanic/mania induced by antidepressants and hypomanic/mania as a phase of bipolar disorder [60]. Fourth, we only used medical records to identify manic and hypomanic episodes and did not investigate other independent sources (e.g., family member accounts), leading to possible case omissions. Fifth, the data on adherence to the use of antidepressants were unavailable. Sixth, patients who were not prescribed antidepressants at the same visit as their bipolar depression diagnosis but received prescriptions at subsequent visits were not included in the AD group. This approach, while adhering to the intention-to-treat principle, may have introduced misclassification bias, potentially leading to an underestimation of the risk associated with antidepressant use. Seventh, a notable limitation of this study is the lack of information on antidepressant dosages across the databases. The absence of a unified method for calculating dose equivalence has posed significant challenges to data standardization and governance, making it difficult to extract and harmonize dosage information. As a result, we were unable to conduct dose–response analyses or evaluate the specific effects of low-dose antidepressants, such as tricyclics and trazodone, which are frequently used for off-label purposes. This limitation restricts our findings to general antidepressant use without accounting for potential dose-related variations in outcomes. Eighth, another limitation of our study is the lack of detailed information on the duration of antidepressant use, which prevents a comprehensive assessment of treatment exposure and its potential impact on outcomes. Ninth, a limitation of this study is that while we focused on patients first diagnosed with bipolar disorder between 2013 January 1 and 2017 December 31, it is possible that some individuals had been diagnosed with bipolar disorder prior to this period. However, our data did not capture any prior diagnoses, which may affect the representativeness of the sample. In addition, our data did not support analysis for exploring the difference in the mean duration of depressive episodes between patients with or without antidepressants, which indicated time to remission as the efficacy of various treatment regimens. Moreover, our study was unable to conduct subgroup analyses to examine the impact of specific antidepressant types or the duration of antidepressant use on the risk of manic switch. These variables, which are well-documented as significant modifiers of manic switch risk, could not be thoroughly evaluated due to limitations in the available data. This limitation highlights the need for cautious interpretation of our findings, as the lack of stratification may affect the precision and generalizability of the results.

## Conclusion

This large-scale retrospective cohort study provided convincing evidence that antidepressants were widely prescribed in clinical settings for the treatment of bipolar depression, including as initial treatment. Furthermore, antidepressant use was not associated with an increased risk of manic/hypomanic switch compared to non-antidepressant treatment. Therefore, antidepressants could be considered a treatment option for bipolar depression. Further studies are needed to explore the risk–benefit ratios in various antidepressant classes and different patient subgroups.

## Data Availability

The authors received permission to access the data used in the study, however, they are unable to share the data as they are not the data custodians. The data from the USA, Germany, and France are owned by IQVIA and reside in IQVIA’s proprietary server domain. The data from China are owned by Beijing Anding Hospital and reside in Beijing Anding Hospital’s proprietary server domain.
