# Supplementary material for: Risk of Switch to Mania/Hypomania in Bipolar Depressive Patients Treated with Antidepressants: A Real-World Study
Source: Health Data Sci. 2025 Jun 3;5:0209. doi: 10.34133/hds.0209 (PMC12130621; doi:10.34133/hds.0209)
Supplement: Supplementary 1 — Figs. S1 to S5 Tables S1 to S9 [file hds.0209.f1.docx]

**Supplementary materials**

**Supplementary Table 1. Data source descriptions**

| **Data Source** | **Description** | **Abbreviation** | **Population, millions** |
| --- | --- | --- | --- |
| IQVIA Open Claims | Pre-adjudicated claims at the anonymized patient level collected from office-based physicians and specialists via office management software and clearinghouse switch sources for the purpose of reimbursement. | Open Claims | 736.3 |
| IQVIA Hospital Charge Data Master | Anonymized patient level data sourced from hospital charge data masters (CDM) and collected from short-term, acute-care and non-federal hospitals. | Hospital CDM | 87.9 |
| IQVIA Disease Analyzer Germany | Disease Analyzer (DA) Germany database consists of data collected from physician and medical centers for all ages. Mostly primary care physician data, some data from specialty practices (where practices are electronically connected to each other) and some lab data is included. Key attributes include demographics, prescriptions, diagnosis, lab measurements, actions (e.g. referrals, sick notes). | DA Germany | 37.6 |
| IQVIA Disease Analyzer France | Disease Analyzer (DA) France database consists of data collected from physician and medical centers for all ages. General practice data is included. Key attributes include demographics, prescriptions, diagnosis, lab measurements, procedures. | DA France | 7.2 |
| Beijing Anding Hospital | Anonymized electronic health records from Electronic Medical (EMR), Laboratory Information System (LIS) and Hospital Information System (HIS). Psychiatric data is included, including populations with mental disorders. | BJ Anding | 0.3 |

**Supplementary Table 2. ICD-10 Diagnosis Codes**

| ICD-10 Code | Description |
| --- | --- |
| Bipolar Disorder Current Episode Depression |  |
| F31.3 | Bipolar affective disorder, current episode mild or moderate depression |
| F31.4 | Bipolar affective disorder, current episode severe depression without psychotic symptoms |
| F31.5 | Bipolar affective disorder, current episode severe depression with psychotic symptoms |
| Mania/hypomania |  |
| F30.0 | Hypomania |
| F30.1 | Mania without psychotic symptoms |
| F30.2 | Mania with psychotic symptoms |
| F30.8 | Other manic episodes |
| F30.9 | Manic episode, unspecified |
| F31.0 | Bipolar affective disorder, current episode hypomanic |
| F31.1 | Bipolar affective disorder, current episode manic without psychotic symptoms |
| F31.2 | Bipolar affective disorder, current episode manic severe with psychotic symptoms |

**Supplementary Table 3. A List of Antidepressants Identified by RxNorm Codes**

| **Concept Id** | **Concept Code** | **Concept Name** | **Domain** | **Standard Concept Caption** | **Class** |
| --- | --- | --- | --- | --- | --- |
| 44507700 | 1455099 | vortioxetine | Drug | Standard | SARIs |
| 19008261 | 11196 | viloxazine | Drug | Standard | other |
| 743670 | 39786 | venlafaxine | Drug | Standard | SNRIs |
| 705755 | 10834 | trimipramine | Drug | Standard | TCAs |
| 703547 | 10737 | trazodone | Drug | Standard | SARIs |
| 703470 | 10734 | tranylcypromine | Drug | Standard | MAOI |
| 19041910 | 38252 | tianeptine | Drug | Standard | other |
| 739138 | 36437 | sertraline | Drug | Standard | SSRIs |
| 19084693 | 60842 | reboxetine | Drug | Standard | NRIs |
| 754270 | 8886 | protriptyline | Drug | Standard | TCAs |
| 733896 | 8123 | phenelzine | Drug | Standard | MAOI |
| 722031 | 32937 | paroxetine | Drug | Standard | SSRIs |
| 721724 | 7531 | nortriptyline | Drug | Standard | TCAs |
| 714684 | 31565 | nefazodone | Drug | Standard | SARIs |
| 19010652 | 30121 | moclobemide | Drug | Standard | MAOI |
| 725131 | 15996 | mirtazapine | Drug | Standard | NaSSA |
| 19080226 | 588250 | milnacipran | Drug | Standard | SNRIs |
| 19007737 | 6929 | mianserin | Drug | Standard | TetraCA |
| 794147 | 6646 | maprotiline | Drug | Standard | TetraCA |
| 19091830 | 6465 | lofepramine | Drug | Standard | TCAs |
| 43560354 | 1433212 | levomilnacipran | Drug | Standard | SNRIs |
| 781705 | 6011 | isocarboxazid | Drug | Standard | MAOI |
| 778268 | 5691 | imipramine | Drug | Standard | TCAs |
| 751412 | 42355 | fluvoxamine | Drug | Standard | SSRIs |
| 755695 | 4493 | fluoxetine | Drug | Standard | SSRIs |
| 715939 | 321988 | escitalopram | Drug | Standard | SSRIs |
| 715259 | 72625 | duloxetine | Drug | Standard | SNRIs |
| 738156 | 3638 | doxepin | Drug | Standard | TCAs |
| 19037989 | 3634 | dothiepin | Drug | Standard | TCAs |
| 19023846 | 3332 | dibenzepin | Drug | Standard | TCAs |
| 717607 | 734064 | desvenlafaxine | Drug | Standard | SNRIs |
| 716968 | 3247 | desipramine | Drug | Standard | TCAs |
| 798834 | 2597 | clomipramine | Drug | Standard | TCAs |
| 797617 | 2556 | citalopram | Drug | Standard | SSRIs |
| 750982 | 42347 | bupropion | Drug | Standard | NDRIs |
| 713109 | 722 | amoxapine | Drug | Standard | TCAs |
| 710062 | 704 | amitriptyline | Drug | Standard | TCAs |
| 21604706 | N06AA19 | amineptine | Drug | Standard | TCAs |
| 36878783 | OMOP997499 | agomelatine | Drug | Standard | MTRA |

Abbreviation: SSRIs, selective serotonin reuptake inhibitors; SNRIs, serotonin–

norepinephrine reuptake inhibitors; SARIs, serotonin antagonist and reuptake inhibitors; NRI, noradrenaline reuptake inhibitor; NaSSA, noradrenergic and specific serotonergic antidepressants; TCAs, tricyclic antidepressants; MAOI, monoamine oxidase inhibitors; TetraCA, tetracyclic antidepressant; NDRI, noradrenaline and dopamine reuptake inhibitor; MTRA, melatonin receptor antagonists.


**Supplementary Table 4. Negative controls to explore residual confounding**

| Concept Set ID | Name | Concept ID | Concept Code | Concept Name | Domain |
| --- | --- | --- | --- | --- | --- |
| 8505 | Bipolar Negative Controls | 376707 | 53726008 | Acute conjunctivitis | Condition |
| 8505 | Bipolar Negative Controls | 257007 | 61582004 | Allergic rhinitis | Condition |
| 8505 | Bipolar Negative Controls | 4067069 | 201040000 | Callosity | Condition |
| 8505 | Bipolar Negative Controls | 380094 | 57406009 | Carpal tunnel syndrome | Condition |
| 8505 | Bipolar Negative Controls | 255573 | 13645005 | Chronic obstructive lung disease | Condition |
| 8505 | Bipolar Negative Controls | 257012 | 40055000 | Chronic sinusitis | Condition |
| 8505 | Bipolar Negative Controls | 134438 | 40275004 | Contact dermatitis | Condition |
| 8505 | Bipolar Negative Controls | 78619 | 22878006 | Contusion of knee | Condition |
| 8505 | Bipolar Negative Controls | 378752 | 64634000 | Corneal opacity | Condition |
| 8505 | Bipolar Negative Controls | 133228 | 80967001 | Dental caries | Condition |
| 8505 | Bipolar Negative Controls | 134681 | 79962008 | Diffuse spasm of esophagus | Condition |
| 8505 | Bipolar Negative Controls | 432251 | 17322007 | Disease caused by parasite | Condition |
| 8505 | Bipolar Negative Controls | 378161 | 25906001 | Disorder of ear | Condition |
| 8505 | Bipolar Negative Controls | 139057 | 41188003 | Disorder of oral soft tissues | Condition |
| 8505 | Bipolar Negative Controls | 31057 | 75860007 | Disorder of pharynx | Condition |
| 8505 | Bipolar Negative Controls | 138225 | 3441005 | Disorder of sebaceous gland | Condition |
| 8505 | Bipolar Negative Controls | 440329 | 111859007 | Herpes zoster without complication | Condition |
| 8505 | Bipolar Negative Controls | 441788 | 240532009 | Human papilloma virus infection | Condition |
| 8505 | Bipolar Negative Controls | 140673 | 40930008 | Hypothyroidism | Condition |
| 8505 | Bipolar Negative Controls | 374375 | 18070006 | Impacted cerumen | Condition |
| 8505 | Bipolar Negative Controls | 139099 | 400097005 | Ingrowing nail | Condition |
| 8505 | Bipolar Negative Controls | 132466 | 209565008 | Lumbar sprain | Condition |
| 8505 | Bipolar Negative Controls | 380733 | 16001004 | Otalgia | Condition |
| 8505 | Bipolar Negative Controls | 372328 | 65363002 | Otitis media | Condition |
| 8505 | Bipolar Negative Controls | 4002650 | 202882003 | Plantar fasciitis | Condition |
| 8505 | Bipolar Negative Controls | 373478 | 41256004 | Presbyopia | Condition |
| 8505 | Bipolar Negative Controls | 438688 | 31541009 | Sarcoidosis | Condition |
| 8505 | Bipolar Negative Controls | 372409 | 23056005 | Sciatica | Condition |
| 8505 | Bipolar Negative Controls | 73562 | 239815007 | Solitary sacroiliitis | Condition |
| 8505 | Bipolar Negative Controls | 133141 | 6020002 | Tinea pedis | Condition |
| 8505 | Bipolar Negative Controls | 436070 | 34713006 | Vitamin D deficiency | Condition |
| 8505 | Bipolar Negative Controls | 201820 | 73211009 | Diabetes mellitus | Condition |
| 8505 | Bipolar Negative Controls | 316866 | 38341003 | Hypertensive disorder | Condition |

**Supplementary Table 5. Baseline Characteristics of Patients with Bipolar Depression Whether Given Antidepressants or Not Before and After Propensity score matching in IQVIA DA France**

| **Characteristic** | **Before Propensity score matching** | | | **After Propensity score matching** | | |
| --- | --- | --- | --- | --- | --- | --- |
|  | **AD** | **Non-AD** |  | **AD** | **Non-AD** |  |
|  | **Percent(%)** | **Percent(%)** | **Std. diff** | **Percent(%)** | **Percent(%)** | **Std. diff** |
| Age group |  |  |  |  |  |  |
| 15 - 19 | <1.0 | 1.2 | -0.12 | <1.4 | <1.4 | -0.07 |
| 20 - 24 | 1.6 | 2.8 | -0.08 | 1.9 | 2.7 | -0.06 |
| 25 - 29 | 4.4 | 4.8 | -0.02 | 3.8 | 4.4 | -0.03 |
| 30 - 34 | 3.6 | 7.2 | -0.16 | 3.8 | 5.8 | -0.09 |
| 35 - 39 | 9.2 | 6.7 | 0.09 | 9 | 7.1 | 0.07 |
| 40 - 44 | 8 | 10.7 | -0.09 | 7.9 | 10.1 | -0.08 |
| 45 - 49 | 16.3 | 13.3 | 0.08 | 17 | 12.9 | 0.12 |
| 50 - 54 | 12 | 16.3 | -0.12 | 14.2 | 15.1 | -0.02 |
| 55 - 59 | 14.7 | 9.1 | 0.17 | 11 | 9.9 | 0.04 |
| 60 - 64 | 9 | 8.7 | 0.01 | 9.3 | 11.5 | -0.07 |
| 65 - 69 | 7.6 | 7.9 | -0.01 | 7.1 | 7.9 | -0.03 |
| 70 - 74 | 6.6 | 4.5 | 0.09 | 6.8 | 5.5 | 0.06 |
| 75 - 79 | 3.6 | 3.2 | 0.02 | 4.4 | 2.7 | 0.09 |
| 80 - 84 | 2.4 | 2.2 | 0.01 | 2.5 | 3 | -0.03 |
| Medical history: General |  |  |  |  |  |  |
| Acute respiratory disease | 13.9 | 13.6 | 0.01 | 12.3 | 13.2 | -0.03 |
| Chronic obstructive lung disease | 1 | 2.2 | -0.1 | <1.4 | 2.5 | -0.1 |
| Diabetes mellitus | 3.4 | 4.7 | -0.07 | 3.6 | 4.1 | -0.03 |
| Gastroesophageal reflux disease | 3.6 | 3.1 | 0.03 | 3 | 3.6 | -0.03 |
| Gastrointestinal hemorrhage | <1.0 | <0.7 | 0.07 | <1.4 | <1.4 | 0.04 |
| Hyperlipidemia | 2.2 | 3.4 | -0.07 | 1.6 | 4.1 | -0.15 |
| Hypertensive disorder | 12 | 9.5 | 0.08 | 11.5 | 11.2 | 0.01 |
| Obesity | 2.4 | 1.8 | 0.04 | 1.9 | <1.4 | 0.07 |
| Osteoarthritis | 5.4 | 4.3 | 0.05 | 5.2 | 4.7 | 0.03 |
| Pneumonia | 1 | <0.7 | 0.07 | <1.4 | <1.4 | 0.07 |
| Psoriasis | <1.0 | 1.8 | -0.09 | <1.4 | 1.6 | -0.07 |
| Renal impairment | <1.0 | <0.7 | 0.05 | <1.4 | <1.4 | 0.04 |
| Urinary tract infectious disease | 1 | 1.5 | -0.04 | <1.4 | <1.4 | 0.03 |
| Visual system disorder | 2.4 | 3.8 | -0.08 | 2.5 | 2.7 | -0.02 |
| Medical history: Cardiovascular disease |  |  |  |  |  |  |
| Cerebrovascular disease | <1.0 | 0.9 | -0.01 | <1.4 | <1.4 | 0.03 |
| Heart disease | 2.4 | 3.5 | -0.07 | 2.2 | 4.9 | -0.15 |
| Heart failure | <1.0 | 0.7 | -0.08 | <1.4 | <1.4 | -0.07 |
| Ischemic heart disease | 1 | 1.6 | -0.05 | <1.4 | 2.5 | -0.13 |
| Medical history: Neoplasms |  |  |  |  |  |  |
| Malignant neoplastic disease | 1.4 | 0.7 | 0.06 | <1.4 | <1.4 | 0.07 |
| Medication use |  |  |  |  |  |  |
| Agents acting on the renin-angiotensin system | 14.1 | 11.6 | 0.08 | 13.2 | 15.1 | -0.06 |
| Antibacterials for systemic use | 35.7 | 37.2 | -0.03 | 34.5 | 35.9 | -0.03 |
| Antiepileptics | 36.9 | 30.6 | 0.13 | 34.2 | 36.7 | -0.05 |
| Antiinflammatory and antirheumatic products | 57.8 | 52.1 | 0.12 | 56.4 | 57.5 | -0.02 |
| Antineoplastic agents | <1.0 | 1.2 | -0.04 | <1.4 | 1.6 | -0.05 |
| Antipsoriatics | 1.2 | 1.3 | -0.01 | 1.4 | <1.4 | 0.05 |
| Beta blocking agents | 13.5 | 10.3 | 0.1 | 11 | 14 | -0.09 |
| Calcium channel blockers | 7.4 | 5.7 | 0.07 | 7.9 | 7.4 | 0.02 |
| Diuretics | 10 | 7 | 0.1 | 9.9 | 6.8 | 0.11 |
| Drugs for acid related disorders | 33.7 | 29 | 0.1 | 31.8 | 35.6 | -0.08 |
| Drugs for obstructive airway diseases | 25.1 | 25.1 | 0 | 25.8 | 25.5 | 0.01 |
| Drugs used in diabetes | 5.8 | 5.3 | 0.02 | 5.5 | 5.2 | 0.01 |
| Immunosuppressants | <1.0 | <0.7 | -0.01 | <1.4 | <1.4 | 0.04 |
| Lipid modifying agents | 14.1 | 14.1 | 0 | 13.4 | 17.5 | -0.11 |
| Opioids | 22.3 | 20.7 | 0.04 | 20.8 | 23.6 | -0.07 |
| Psycholeptics | 84.1 | 65.1 | 0.45 | 78.1 | 80 | -0.05 |
| Psychostimulants, agents used for ADHD and nootropics | 4.2 | 5.1 | -0.04 | 4.4 | 6 | -0.07 |

Abbreviations: AD, antidepressants; Std. diff, standardized difference.

**Supplementary Table 6. Baseline Characteristics of Patients with Bipolar Depression Whether Given Antidepressants or Not Before and After Propensity score matching in IQVIA DA Germany**

| **Characteristic** | **Before Propensity score matching** | | | **After Propensity score matching** | | |
| --- | --- | --- | --- | --- | --- | --- |
|  | **AD** | **Non-AD** |  | **AD** | **Non-AD** |  |
|  | **Percent(%)** | **Percent(%)** | **Std. diff** | **Percent(%)** | **Percent(%)** | **Std. diff** |
| Age group |  |  |  |  |  |  |
| 15 - 19 | 0.8 | 1.8 | -0.09 | 0.9 | 2.3 | -0.11 |
| 20 - 24 | 2.7 | 4.1 | -0.08 | 2.9 | 3.7 | -0.04 |
| 25 - 29 | 4.2 | 5.6 | -0.07 | 4.6 | 6.2 | -0.07 |
| 30 - 34 | 4.1 | 6.5 | -0.11 | 4.5 | 6 | -0.07 |
| 35 - 39 | 8.3 | 6.8 | 0.06 | 8.5 | 7.5 | 0.04 |
| 40 - 44 | 7.5 | 7 | 0.02 | 7 | 7.1 | 0 |
| 45 - 49 | 11.2 | 9.6 | 0.05 | 11.8 | 8.4 | 0.11 |
| 55 - 59 | 14.8 | 13.4 | 0.04 | 14.6 | 14.5 | 0 |
| 60 - 64 | 9.8 | 10.6 | -0.03 | 9.6 | 11.1 | -0.05 |
| 65 - 69 | 6.3 | 5.7 | 0.03 | 6.4 | 5.9 | 0.02 |
| 70 - 74 | 6.3 | 5 | 0.06 | 6 | 4.9 | 0.05 |
| 75 - 79 | 5.3 | 5.2 | 0.01 | 5 | 4.2 | 0.04 |
| 80 - 84 | 2.6 | 3.7 | -0.06 | 2.3 | 2.7 | -0.03 |
| 85 - 89 | 1.4 | 1.1 | 0.02 | 1.3 | 1.1 | 0.02 |
| 90 - 94 | <0.5 | 0.3 | 0 | <0.5 | <0.5 | 0.02 |
| Gender: female | 61.7 | 61.5 | 0.01 | 61.1 | 59.7 | 0.03 |
| Medical history: General |  |  |  |  |  |  |
| Acute respiratory disease | 4.8 | 10.6 | -0.22 | 5.5 | 4.5 | 0.04 |
| Chronic obstructive lung disease | 0.7 | 1.8 | -0.09 | 0.9 | 1.1 | -0.02 |
| Crohn's disease | <0.5 | <0.2 | -0.02 | <0.5 | <0.5 | -0.03 |
| Dementia | 0.7 | 0.6 | 0.01 | 0.5 | 0.6 | -0.01 |
| Diabetes mellitus | 2.8 | 4.8 | -0.1 | 2.9 | 2.5 | 0.03 |
| Gastroesophageal reflux disease | <0.5 | 1.5 | -0.12 | <0.5 | 1 | -0.08 |
| Gastrointestinal hemorrhage | <0.5 | 0.6 | -0.04 | <0.5 | <0.5 | 0.06 |
| Human immunodeficiency virus infection | <0.5 | <0.2 | 0.01 | <0.5 | <0.5 | -0.03 |
| Hyperlipidemia | 2.1 | 4.8 | -0.15 | 2.1 | 2 | 0.01 |
| Hypertensive disorder | 6.2 | 11.1 | -0.17 | 6.9 | 5.3 | 0.07 |
| Obesity | 2.2 | 5.5 | -0.17 | 2.3 | 2.5 | -0.01 |
| Osteoarthritis | 2.3 | 8.2 | -0.27 | 2.7 | 2 | 0.04 |
| Renal impairment | 0.5 | 1.6 | -0.11 | 0.5 | 0.6 | -0.01 |
| Rheumatoid arthritis | 0.6 | 0.9 | -0.03 | 0.8 | <0.5 | 0.08 |
| Schizophrenia | <0.5 | 0.4 | -0.04 | <0.5 | 0.6 | -0.07 |
| Urinary tract infectious disease | 0.7 | 1.5 | -0.07 | 0.6 | 0.9 | -0.03 |
| Visual system disorder | 1.9 | 3.4 | -0.1 | 2 | 1.6 | 0.03 |
| Medical history: Cardiovascular disease |  |  |  |  |  |  |
| Atrial fibrillation | <0.5 | 0.7 | -0.1 | <0.5 | <0.5 | -0.03 |
| Cerebrovascular disease | 1.6 | 1.6 | 0 | 1.5 | 1.4 | 0.01 |
| Coronary arteriosclerosis | <0.5 | 0.7 | -0.08 | <0.5 | <0.5 | -0.02 |
| Heart disease | 3.7 | 5.8 | -0.1 | 4 | 2.9 | 0.06 |
| Heart failure | 1.1 | 1.1 | 0 | 1.2 | 0.9 | 0.03 |
| Ischemic heart disease | 1.4 | 1.9 | -0.04 | 1.3 | 0.8 | 0.05 |
| Medical history: Neoplasms |  |  |  |  |  |  |
| Malignant neoplastic disease | 0.7 | 1.8 | -0.09 | 0.8 | 1.1 | -0.03 |
| Malignant tumor of breast | <0.5 | 0.3 | 0 | <0.5 | <0.5 | 0.02 |
| Medication use |  |  |  |  |  |  |
| Agents acting on the renin-angiotensin system | 5.2 | 11.1 | -0.22 | 5.9 | 5.3 | 0.03 |
| Antibacterials for systemic use | 5.2 | 10.6 | -0.2 | 5.9 | 3.7 | 0.11 |
| Antiepileptics | 26.1 | 15.5 | 0.26 | 24.5 | 26 | -0.04 |
| Antiinflammatory and antirheumatic products | 7.6 | 14.2 | -0.21 | 8.1 | 7.3 | 0.03 |
| Antineoplastic agents | <0.5 | 0.7 | -0.08 | <0.5 | <0.5 | -0.04 |
| Antithrombotic agents | 2.9 | 5 | -0.11 | 3.2 | 2.3 | 0.06 |
| Beta blocking agents | 3.7 | 7.9 | -0.18 | 4.2 | 3.1 | 0.06 |
| Calcium channel blockers | 2.4 | 4.6 | -0.12 | 2.7 | 1.8 | 0.06 |
| Diuretics | 3.3 | 7.6 | -0.19 | 3.9 | 3.7 | 0.01 |
| Drugs for acid related disorders | 7.1 | 11.4 | -0.15 | 7.8 | 7.1 | 0.03 |
| Drugs used in diabetes | 2 | 3.5 | -0.09 | 2.3 | 1.3 | 0.07 |
| Immunosuppressants | 0.7 | 0.3 | 0.06 | <0.5 | <0.5 | 0.02 |
| Lipid modifying agents | 2.8 | 5.4 | -0.13 | 3.1 | 2.4 | 0.05 |
| Opioids | 3.6 | 5.7 | -0.1 | 3.5 | 3.9 | -0.02 |
| Psycholeptics | 50.9 | 38.6 | 0.25 | 51.3 | 55.4 | -0.08 |
| Psychostimulants, agents used for ADHD and nootropics | 1.4 | 1.1 | 0.03 | 1.5 | 1.7 | -0.02 |

Abbreviations: AD, antidepressants; Std. diff, standardized difference.

**Supplementary Table 7. Baseline Characteristics of Patients with Bipolar Depression Whether Given Antidepressants or Not Before and After Propensity score matching in IQVIA US Hospital CDM**

| **Characteristic** | **Before Propensity score matching** | | | **After Propensity score matching** | | |
| --- | --- | --- | --- | --- | --- | --- |
|  | **AD** | **Non-AD** |  | **AD** | **Non-AD** |  |
|  | **Percent(%)** | **Percent(%)** | **Std. diff** | **Percent(%)** | **Percent(%)** | **Std. diff** |
| Age group |  |  |  |  |  |  |
| 5 - 9 | <0.2 | 0.1 | -0.02 | <0.2 | <0.2 | -0.02 |
| 10 -14 | 0.2 | 0.4 | -0.05 | 0.2 | <0.2 | 0.01 |
| 15 - 19 | 1.4 | 2.4 | -0.07 | 1.6 | 1.7 | -0.01 |
| 20 - 24 | 3.9 | 6.3 | -0.11 | 4.1 | 4.9 | -0.04 |
| 25 - 29 | 5.3 | 7.9 | -0.1 | 5.6 | 6.1 | -0.02 |
| 30 - 34 | 7 | 9.4 | -0.09 | 7.3 | 7.7 | -0.01 |
| 35 - 39 | 8.1 | 9.6 | -0.05 | 8.3 | 8.5 | -0.01 |
| 40 - 44 | 9.7 | 10.1 | -0.01 | 9.8 | 10.4 | -0.02 |
| 45 - 49 | 11.8 | 10.4 | 0.05 | 11.7 | 11 | 0.03 |
| 50 - 54 | 14.4 | 12.2 | 0.06 | 13.9 | 13.7 | 0 |
| 60 - 64 | 9.7 | 8.1 | 0.05 | 9.5 | 9.1 | 0.01 |
| 65 - 69 | 7.7 | 5.8 | 0.08 | 7.4 | 6.8 | 0.02 |
| 70 - 74 | 4.6 | 3.2 | 0.07 | 4.5 | 4.3 | 0.01 |
| 75 - 79 | 2.5 | 1.8 | 0.05 | 2.4 | 2.2 | 0.01 |
| 80 - 84 | 0.7 | 0.6 | 0.01 | 0.7 | 0.4 | 0.03 |
| Gender: female | 73.3 | 70.7 | 0.06 | 73 | 74.1 | -0.03 |
| Medical history: General |  |  |  |  |  |  |
| Acute respiratory disease | 11.8 | 8 | 0.13 | 10.3 | 10.8 | -0.02 |
| Attention deficit hyperactivity disorder | 1.6 | 1.8 | -0.02 | 1.5 | 1.9 | -0.03 |
| Chronic liver disease | 5 | 2.7 | 0.12 | 4.5 | 4.1 | 0.02 |
| Chronic obstructive lung disease | 14 | 5.5 | 0.29 | 11.3 | 11.8 | -0.02 |
| Crohn's disease | 0.9 | 0.3 | 0.07 | 0.7 | 0.5 | 0.03 |
| Dementia | 0.7 | 0.3 | 0.06 | 0.4 | 0.3 | 0.02 |
| Diabetes mellitus | 21.7 | 13.6 | 0.22 | 20 | 20.8 | -0.02 |
| Gastroesophageal reflux disease | 26.8 | 14.1 | 0.32 | 23.9 | 24.3 | -0.01 |
| Gastrointestinal hemorrhage | 3 | 1.4 | 0.11 | 2.8 | 2.9 | -0.01 |
| Human immunodeficiency virus infection | 1.4 | 2 | -0.04 | 1.4 | 1.5 | -0.01 |
| Hyperlipidemia | 31 | 21.5 | 0.22 | 29 | 30.6 | -0.03 |
| Hypertensive disorder | 40.2 | 27.1 | 0.28 | 36.8 | 39.4 | -0.05 |
| Lesion of liver | 2.1 | 1.1 | 0.08 | 1.8 | 2 | -0.01 |
| Obesity | 20.9 | 14.2 | 0.17 | 19.5 | 19.9 | -0.01 |
| Osteoarthritis | 19.3 | 12.2 | 0.19 | 17.9 | 18.7 | -0.02 |
| Pneumonia | 2.7 | 1.1 | 0.11 | 2 | 2.3 | -0.01 |
| Renal impairment | 8.9 | 4 | 0.2 | 7.3 | 7 | 0.01 |
| Rheumatoid arthritis | 2.6 | 1.2 | 0.1 | 2.1 | 2.3 | -0.02 |
| Urinary tract infectious disease | 9.5 | 5.3 | 0.16 | 8.1 | 8.3 | -0.01 |
| Viral hepatitis C | 4.2 | 2.7 | 0.08 | 3.9 | 3.3 | 0.03 |
| Visual system disorder | 6.8 | 5.8 | 0.04 | 6.4 | 7.5 | -0.04 |
| Medical history: Cardiovascular disease |  |  |  |  |  |  |
| Atrial fibrillation | 2.9 | 1.2 | 0.12 | 2.3 | 2.3 | 0 |
| Cerebrovascular disease | 1.9 | 0.8 | 0.1 | 1.5 | 1.6 | -0.01 |
| Coronary arteriosclerosis | 8.2 | 3.6 | 0.2 | 6.6 | 7.6 | -0.04 |
| Heart disease | 20.1 | 9.5 | 0.3 | 16.6 | 17 | -0.01 |
| Heart failure | 5 | 2 | 0.17 | 3.7 | 4.4 | -0.04 |
| Ischemic heart disease | 5 | 2 | 0.17 | 4.1 | 3.9 | 0.01 |
| Peripheral vascular disease | 2.6 | 1.3 | 0.1 | 2.1 | 2.1 | 0 |
| Pulmonary embolism | 0.9 | 0.3 | 0.08 | 0.7 | 0.4 | 0.03 |
| Venous thrombosis | 1.5 | 0.6 | 0.09 | 1.2 | 1.1 | 0.01 |
| Medical history: Neoplasms |  |  |  |  |  |  |
| Hematologic neoplasm | 0.7 | 0.5 | 0.03 | 0.6 | 0.9 | -0.03 |
| Malignant lymphoma | 0.3 | 0.2 | 0.02 | 0.2 | 0.3 | -0.01 |
| Malignant neoplasm of anorectum | 0.2 | 0.1 | 0.02 | 0.2 | <0.2 | 0.01 |
| Malignant neoplastic disease | 5.9 | 3.4 | 0.12 | 5.7 | 5.6 | 0.01 |
| Malignant tumor of colon | 0.4 | 0.2 | 0.04 | 0.3 | 0.3 | 0.01 |
| Malignant tumor of lung | 0.4 | 0.2 | 0.03 | 0.3 | 0.2 | 0.01 |
| Malignant tumor of urinary bladder | 0.2 | 0.1 | 0.01 | <0.2 | 0.3 | -0.03 |
| Primary malignant neoplasm of prostate | 0.4 | 0.2 | 0.02 | 0.4 | 0.4 | 0.01 |
| Medication use |  |  |  |  |  |  |
| Agents acting on the renin-angiotensin system | 8.1 | 2.2 | 0.27 | 5.5 | 6.3 | -0.04 |
| Antibacterials for systemic use | 30.5 | 14.5 | 0.39 | 26.4 | 28.3 | -0.04 |
| Antiepileptics | 25.2 | 7.4 | 0.5 | 18.4 | 20.9 | -0.06 |
| Antiinflammatory and antirheumatic products | 31.7 | 15.8 | 0.38 | 27.4 | 29.9 | -0.06 |
| Antineoplastic agents | 2.1 | 0.9 | 0.1 | 1.9 | 2.1 | -0.02 |
| Antithrombotic agents | 24.2 | 7.6 | 0.46 | 18 | 19.8 | -0.05 |
| Beta blocking agents | 13.5 | 3.9 | 0.35 | 9.5 | 11.6 | -0.07 |
| Calcium channel blockers | 6.8 | 1.8 | 0.25 | 4.6 | 5.2 | -0.03 |
| Diuretics | 8.1 | 1.9 | 0.29 | 5.1 | 6.1 | -0.04 |
| Drugs for acid related disorders | 25.4 | 8.4 | 0.47 | 19.5 | 21.4 | -0.05 |
| Drugs for obstructive airway diseases | 28.5 | 13 | 0.39 | 24.3 | 25.6 | -0.03 |
| Drugs used in diabetes | 6.2 | 1.6 | 0.24 | 4.3 | 4.9 | -0.03 |
| Immunosuppressants | 0.9 | 0.3 | 0.08 | 0.6 | 0.5 | 0 |
| Lipid modifying agents | 10.1 | 2.5 | 0.32 | 7 | 7.9 | -0.04 |
| Opioids | 39.1 | 19.1 | 0.45 | 34.4 | 36.8 | -0.05 |
| Psycholeptics | 45.4 | 21 | 0.54 | 39.4 | 43.8 | -0.09 |
| Psychostimulants, agents used for ADHD and nootropics | 2.4 | 0.6 | 0.15 | 1.8 | 1.6 | 0.01 |

Abbreviations: AD, antidepressants; Std. diff, standardized difference.

**Supplementary Table 8. Baseline Characteristics of Patients with Bipolar Depression Whether Given Antidepressants or Not Before and After Propensity score matching in IQVIA US Open Claims**

| **Characteristic** | **Before Propensity score matching** | | | **After Propensity score matching** | | |
| --- | --- | --- | --- | --- | --- | --- |
|  | **AD** | **Non-AD** |  | **AD** | **Non-AD** |  |
|  | **Percent(%)** | **Percent(%)** | **Std. diff** | **Percent(%)** | **Percent(%)** | **Std. diff** |
| Age group |  |  |  |  |  |  |
| 0 - 4 | 0.1 | 0.1 | 0 | 0.1 | 0.1 | 0 |
| 5 - 9 | 0.2 | 0.3 | -0.02 | 0.2 | 0.3 | -0.02 |
| 10-14 | 0.9 | 1.1 | -0.02 | 1.3 | 1.2 | 0.01 |
| 15 - 19 | 4.2 | 4.4 | -0.01 | 5.2 | 5.1 | 0 |
| 20 - 24 | 7 | 8.1 | -0.04 | 8.7 | 8.9 | -0.01 |
| 25 - 29 | 8 | 8.6 | -0.02 | 9.6 | 9.6 | 0 |
| 30 - 34 | 9.2 | 8.6 | 0.02 | 9.7 | 9.6 | 0 |
| 35 - 39 | 9.8 | 8.6 | 0.04 | 9.4 | 9.6 | -0.01 |
| 40 - 44 | 9.6 | 7.1 | 0.09 | 8.1 | 8 | 0 |
| 45 - 49 | 10.3 | 7.7 | 0.09 | 8.7 | 8.5 | 0.01 |
| 50 - 54 | 10.9 | 8.1 | 0.1 | 9.1 | 8.7 | 0.01 |
| 55 - 59 | 9.9 | 8.6 | 0.04 | 8.4 | 8.5 | -0.01 |
| 60 - 64 | 7.8 | 8 | -0.01 | 7.1 | 7.2 | 0 |
| 65 - 69 | 5.4 | 7.4 | -0.08 | 5.8 | 5.8 | 0 |
| 70 - 74 | 3.3 | 5.2 | -0.09 | 3.8 | 3.6 | 0.01 |
| 75 - 79 | 2.4 | 4.4 | -0.11 | 3 | 3.1 | 0 |
| 80 - 84 | 1.1 | 3.5 | -0.16 | 1.9 | 2.1 | -0.01 |
| Gender: female | 71.4 | 60.4 | 0.23 | 63.4 | 63.9 | -0.01 |
| Medical history: General |  |  |  |  |  |  |
| Acute respiratory disease | 18 | 14.1 | 0.11 | 14.3 | 14.7 | -0.01 |
| Attention deficit hyperactivity disorder | 2 | 1.7 | 0.02 | 1.9 | 1.9 | 0 |
| Chronic liver disease | 2.1 | 1.8 | 0.02 | 1.7 | 1.8 | 0 |
| Chronic obstructive lung disease | 7.4 | 8.3 | -0.03 | 6.8 | 6.9 | 0 |
| Crohn's disease | 0.4 | 0.3 | 0.02 | 0.2 | 0.3 | -0.02 |
| Dementia | 0.3 | 1.3 | -0.11 | 0.6 | 0.6 | -0.01 |
| Diabetes mellitus | 13.3 | 14.2 | -0.03 | 11.9 | 12.1 | -0.01 |
| Gastroesophageal reflux disease | 9.3 | 7.1 | 0.08 | 6.8 | 6.9 | 0 |
| Gastrointestinal hemorrhage | 2.1 | 2.2 | -0.01 | 2 | 2 | 0 |
| Human immunodeficiency virus infection | 0.7 | 0.7 | 0 | 0.7 | 0.7 | 0 |
| Hyperlipidemia | 17.5 | 14.7 | 0.07 | 14.2 | 14.3 | 0 |
| Hypertensive disorder | 24.2 | 23.9 | 0.01 | 21.2 | 21.5 | -0.01 |
| Lesion of liver | 0.8 | 1.1 | -0.03 | 0.9 | 1 | -0.01 |
| Obesity | 9.6 | 7.7 | 0.07 | 7.9 | 7.8 | 0 |
| Osteoarthritis | 17.1 | 12.7 | 0.12 | 12.4 | 12.4 | 0 |
| Pneumonia | 3.3 | 4.9 | -0.08 | 3.6 | 3.8 | -0.01 |
| Psoriasis | 0.7 | 0.5 | 0.03 | 0.6 | 0.5 | 0.01 |
| Renal impairment | 5.2 | 7.8 | -0.11 | 6 | 5.9 | 0 |
| Rheumatoid arthritis | 1.3 | 0.9 | 0.04 | 0.9 | 0.8 | 0.01 |
| Ulcerative colitis | 0.3 | 0.2 | 0.02 | 0.3 | 0.2 | 0.01 |
| Urinary tract infectious disease | 9 | 8.9 | 0 | 7.9 | 7.9 | 0 |
| Visual system disorder | 12.5 | 11.3 | 0.04 | 11.2 | 11 | 0.01 |
| Medical history: Cardiovascular disease |  |  |  |  |  |  |
| Atrial fibrillation | 1.8 | 3.4 | -0.1 | 2.4 | 2.3 | 0 |
| Cerebrovascular disease | 2.4 | 3.2 | -0.04 | 2.4 | 2.6 | -0.02 |
| Coronary arteriosclerosis | 3.8 | 5.1 | -0.06 | 4 | 3.9 | 0.01 |
| Heart failure | 3.2 | 5.5 | -0.11 | 3.7 | 3.9 | -0.01 |
| Ischemic heart disease | 2.7 | 3.5 | -0.04 | 2.8 | 2.8 | 0 |
| Peripheral vascular disease | 2.6 | 4.6 | -0.11 | 3 | 3 | 0 |
| Pulmonary embolism | 0.7 | 0.8 | -0.01 | 0.7 | 0.6 | 0.01 |
| Venous thrombosis | 1 | 1.3 | -0.02 | 1.1 | 1.1 | 0 |
| Medical history: Neoplasms |  |  |  |  |  |  |
| Hematologic neoplasm | 0.5 | 0.7 | -0.02 | 0.6 | 0.6 | 0 |
| Malignant lymphoma | 0.3 | 0.3 | 0 | 0.3 | 0.2 | 0.02 |
| Malignant neoplasm of anorectum | 0.1 | 0.1 | -0.01 | 0.1 | 0.1 | 0.01 |
| Malignant neoplastic disease | 3.6 | 4 | -0.02 | 3.6 | 3.5 | 0 |
| Malignant tumor of breast | 0.8 | 0.6 | 0.02 | 0.7 | 0.6 | 0.01 |
| Malignant tumor of colon | 0.2 | 0.2 | -0.01 | 0.2 | 0.2 | 0 |
| Malignant tumor of lung | 0.1 | 0.3 | -0.03 | 0.2 | 0.2 | 0 |
| Malignant tumor of urinary bladder | 0.1 | 0.1 | -0.01 | 0.1 | 0.1 | 0.01 |
| Primary malignant neoplasm of prostate | 0.2 | 0.4 | -0.02 | 0.3 | 0.3 | 0 |
| Medication use |  |  |  |  |  |  |
| Agents acting on the renin-angiotensin system | 19.4 | 10.3 | 0.26 | 12.1 | 12.5 | -0.01 |
| Antibacterials for systemic use | 59.9 | 33.5 | 0.55 | 41.3 | 42.3 | -0.02 |
| Antiepileptics | 60.7 | 27.6 | 0.71 | 35.7 | 36.3 | -0.01 |
| Antiinflammatory and antirheumatic products | 38.5 | 19.7 | 0.42 | 24.3 | 25.1 | -0.02 |
| Antineoplastic agents | 4.2 | 2.1 | 0.12 | 2.6 | 2.5 | 0.01 |
| Antipsoriatics | 0.6 | 0.4 | 0.02 | 0.4 | 0.5 | -0.01 |
| Beta blocking agents | 17.5 | 9.3 | 0.24 | 11.1 | 11.3 | 0 |
| Calcium channel blockers | 9.6 | 5.9 | 0.14 | 6.7 | 7 | -0.01 |
| Diuretics | 16.7 | 8.6 | 0.24 | 10.2 | 10.4 | -0.01 |
| Drugs for acid related disorders | 28.6 | 12.6 | 0.4 | 15.4 | 15.9 | -0.01 |
| Drugs for obstructive airway diseases | 37.5 | 18.8 | 0.43 | 23.4 | 23.7 | -0.01 |
| Drugs used in diabetes | 13.2 | 7.4 | 0.19 | 8.6 | 8.8 | -0.01 |
| Immunosuppressants | 2.4 | 1.1 | 0.1 | 1.4 | 1.3 | 0.01 |
| Lipid modifying agents | 22.4 | 11 | 0.31 | 13.3 | 13.4 | 0 |
| Opioids | 37.1 | 18.3 | 0.43 | 22 | 23.3 | -0.03 |
| Psycholeptics | 71.4 | 36 | 0.76 | 45.8 | 47 | -0.02 |
| Psychostimulants, agents used for ADHD and nootropics | 11.1 | 3.9 | 0.28 | 5.2 | 5.4 | -0.01 |

Abbreviations: AD, antidepressants; Std. diff, standardized difference.

**Supplementary Table 9. Baseline Characteristics of Patients with Bipolar Depression Whether Given Antidepressants or Not Before and After Propensity score matching in Bejing Anding Hospital**

| **Characteristic** | **Before Propensity score matching** | | | **After Propensity score matching** | | |
| --- | --- | --- | --- | --- | --- | --- |
|  | **AD** | **Non-AD** |  | **AD** | **Non-AD** |  |
|  | **Percent(%)** | **Percent(%)** | **Std. diff** | **Percent(%)** | **Percent(%)** | **Std. diff** |
| Age group |  |  |  |  |  |  |
| 10-14 | <1.0 | 1.3 | -0.05 | <1.9 | <1.9 | -0.03 |
| 15 - 19 | 9.6 | 11.6 | -0.06 | 10.9 | 9.3 | 0.05 |
| 20 - 24 | 10.2 | 15.4 | -0.16 | 10.9 | 12.8 | -0.06 |
| 25 - 29 | 14.7 | 15.9 | -0.03 | 15.6 | 18.3 | -0.07 |
| 30 - 34 | 12.4 | 11.3 | 0.03 | 9.3 | 12.1 | -0.09 |
| 35 - 39 | 9.4 | 9.8 | -0.01 | 10.5 | 10.1 | 0.01 |
| 50 - 54 | 8.2 | 5.4 | 0.11 | 7.8 | 5.4 | 0.09 |
| 55 - 59 | 7.6 | 6.2 | 0.06 | 7.8 | 7 | 0.03 |
| 60 - 64 | 6.6 | 4.6 | 0.09 | 5.8 | 4.7 | 0.05 |
| 65 - 69 | 2.6 | 3.3 | -0.04 | 1.9 | 3.1 | -0.07 |
| 70 - 74 | 1.8 | <1.3 | 0.12 | 2.3 | <1.9 | 0.17 |
| 75 - 79 | 2 | <1.3 | 0.13 | <1.9 | <1.9 | 0.07 |
| Gender: female | 58.8 | 57.3 | 0.03 | 55.6 | 58 | -0.05 |
| Medical history: General |  |  |  |  |  |  |
| Hyperlipidemia | 1 | <1.3 | 0.06 | <1.9 | <1.9 | 0.04 |
| Hypertensive disorder | 1.2 | <1.3 | 0.07 | <1.9 | <1.9 | 0.04 |
| Medical history: Cardiovascular disease |  |  |  |  |  |  |
| Heart disease | <1.0 | <1.3 | 0.07 | <1.9 | <1.9 | 0.12 |
| Medication use |  |  |  |  |  |  |
| Antibacterials for systemic use | <1.0 | <1.3 | -0.02 | <1.9 | <1.9 | 0.05 |
| Antiepileptics | 59.6 | 52.4 | 0.14 | 54.9 | 63 | -0.17 |
| Beta blocking agents | 3.6 | 3.3 | 0.01 | 3.5 | 2.7 | 0.04 |
| Calcium channel blockers | <1.0 | 1.3 | -0.05 | <1.9 | <1.9 | -0.04 |
| Drugs used in diabetes | 1 | <1.3 | 0.06 | <1.9 | <1.9 | 0.07 |
| Opioids | 1.4 | 3.1 | -0.11 | <1.9 | 2.7 | -0.08 |
| Psycholeptics | 86.1 | 74 | 0.3 | 83.3 | 85.2 | -0.05 |
| Psychostimulants, agents used for ADHD and nootropics | 2.6 | 1.5 | 0.07 | 1.9 | <1.9 | 0.03 |

Abbreviations: AD, antidepressants; Std. diff, standardized difference.


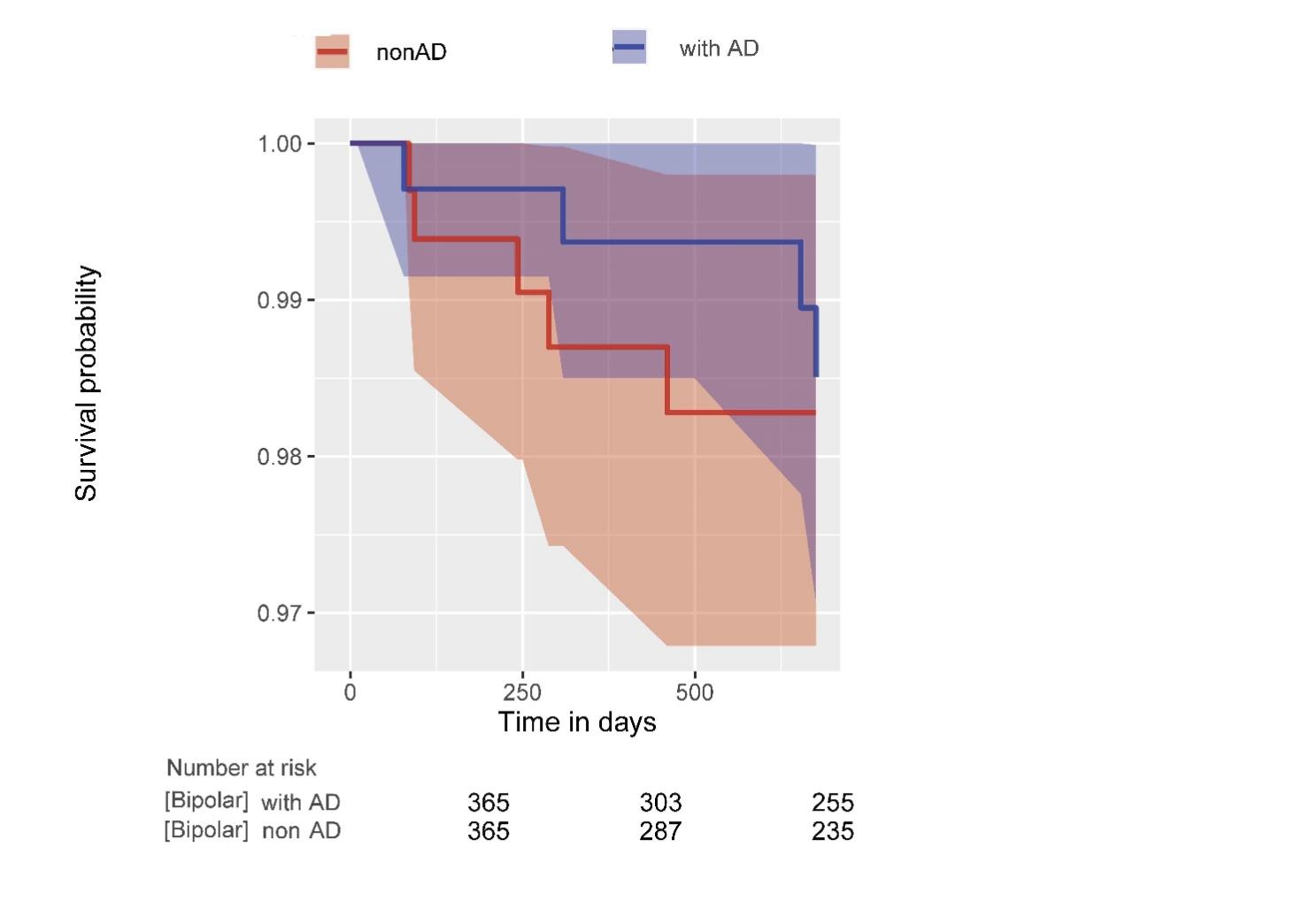


Supplementary Figure 1 Kaplan-Meier curve in IQVIA DA France


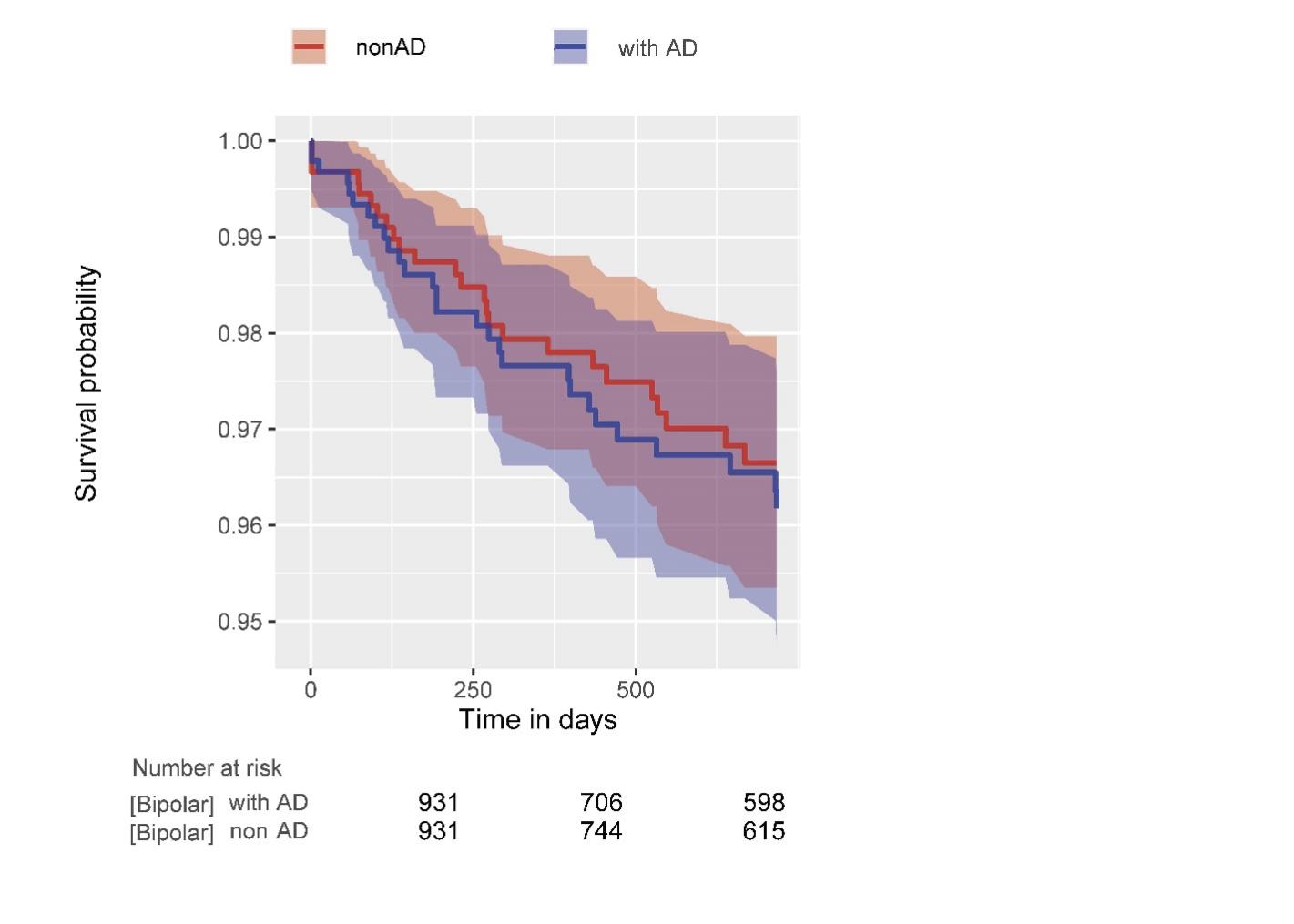


Supplementary Figure 2 Kaplan-Meier curve in IQVIA DA Germany


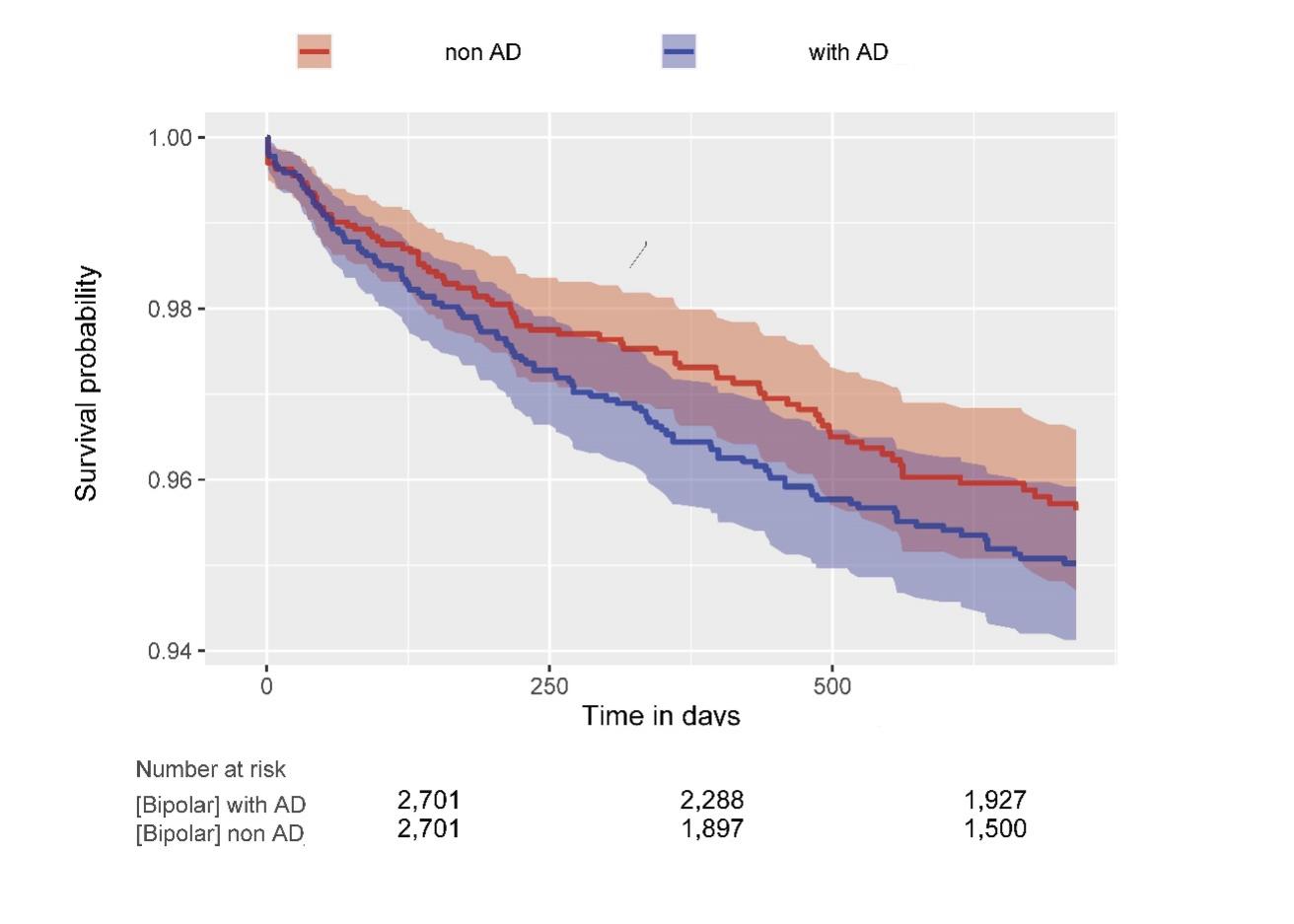


Supplementary Figure 3 Kaplan-Meier curve in IQVIA US Hospital CDM


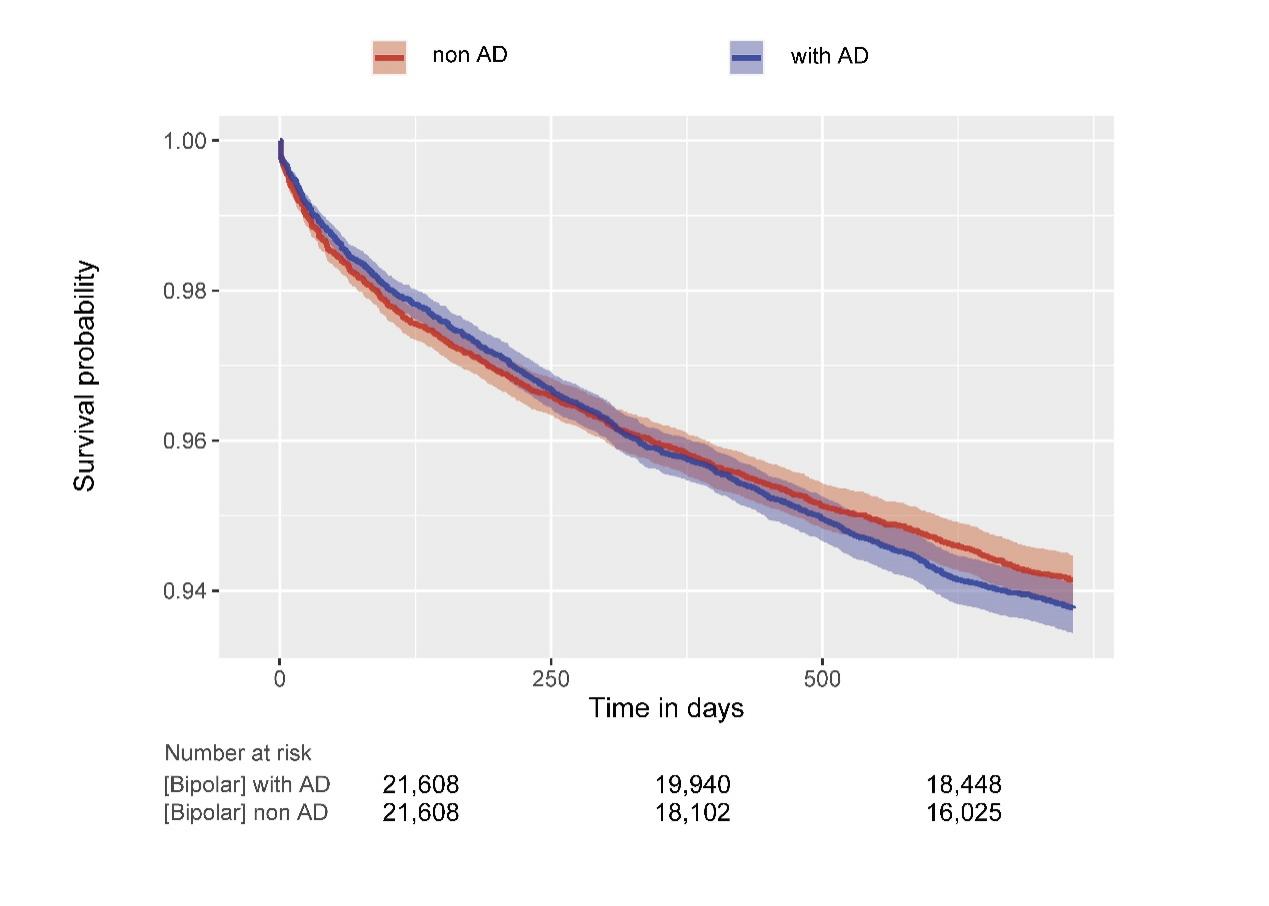


Supplementary Figure 4 Kaplan-Meier curve in IQVIA US Open Claims


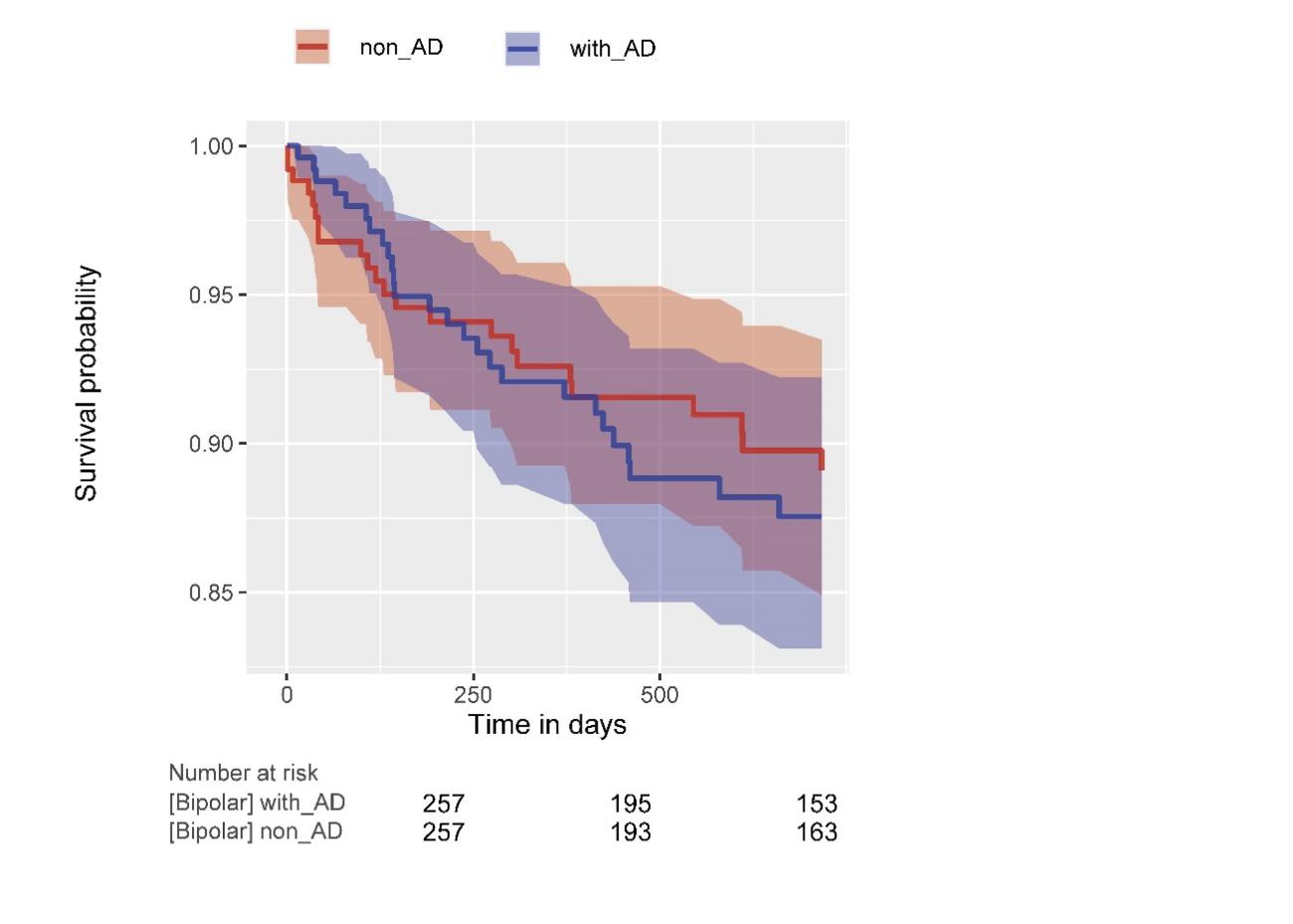


Supplementary Figure 5 Kaplan-Meier curve in Bejing Anding Hospital
